# Development of a Chief Resident Medical Procedure Service: a 10-Year Experience

**DOI:** 10.1007/s11606-023-08234-z

**Published:** 2023-05-26

**Authors:** Robert Nathanson, Hasan Baher, Jason Phillips, Megan Freeman, Raj Sehgal, Jane O’Rorke, Nilam J. Soni

**Affiliations:** 1 Medicine Service, South Texas Veterans Health Care System, San Antonio, TX USA; 2 Division of Hospital Medicine, Department of Medicine, University of Texas Health San Antonio, San Antonio, TX USA; 3 Department of Medicine, University of Texas Health San Antonio, San Antonio, TX USA; 4 Division of Cardiology, University of Texas Health San Antonio, San Antonio, TX USA; 5 Division of General Internal Medicine, University of Texas Health San Antonio, San Antonio, TX USA; 6 Division of Pulmonary Diseases & Critical Care Medicine, University of Texas Health San Antonio, San Antonio, TX USA

**Keywords:** education, point of care, ultrasound, procedures.

## Abstract

**Background:**

Lack of experienced faculty to supervise internal medicine (IM) residents is a significant barrier to establishing a medical procedure service (MPS).

**Aim:**

Describe the development and 10-year outcomes of an MPS led by IM chief residents.

**Setting:**

University-based IM residency program affiliated with a county and Veterans Affairs hospital.

**Participants:**

Categorical IM interns (*n*=320) and 4^th^-year IM chief residents (*n*=48) from 2011 to 2022.

**Program Description:**

The MPS operated on weekdays, 8 am–5 pm. After training and sign-off by the MPS director, chief residents trained and supervised interns in ultrasound-guided procedures during a 4-week rotation.

**Program Evaluation:**

From 2011 to 2022, our MPS received 5967 consults and 4465 (75%) procedures were attempted. Overall procedure success, complication, and major complication rates were 94%, 2.6%, and 0.6%, respectively. Success and complication rates for paracentesis (*n*=2285) were 99% and 1.1%, respectively; 99% and 4.2% for thoracentesis (*n*=1167); 76% and 4.5% for lumbar puncture (*n*=883); 83% and 1.2% for knee arthrocentesis (*n*=85); and 76% and 0% for central venous catheterization (*n*=45). The rotation was rated 4.6 out of 5 for overall learning quality.

**Discussion:**

A chief resident–led MPS is a practical and safe approach for IM residency programs to establish an MPS when experienced attending physicians are unavailable.

**Supplementary Information:**

The online version contains supplementary material available at 10.1007/s11606-023-08234-z.

## INTRODUCTION

Since first described in 2004^1^, several institutions have implemented a medical procedure service (MPS) to improve timeliness of procedures and standardize training of procedures for internal medicine (IM) residents.^2,3^ With the traditional MPS model, residents perform non-urgent bedside procedures under the supervision of an attending physician.^3^ However, a critical barrier to starting an MPS has been a lack of experienced attending physicians with expertise in ultrasound-guided procedures.^4^

In 2011, as a quality improvement initiative for the Department of Veterans Affairs’ Chief Resident in Quality and Patient Safety (CRQS) program, our IM residency program created an MPS led by 4^th^-year IM chief residents with junior faculty appointments. Few studies have evaluated the impact of MPS’s, and none describe an MPS led by chief residents.^3^ Here we describe the development of an MPS led by 4^th^-year IM chief residents and the 10-year outcomes.

## SETTING AND PARTICIPANTS

The MPS was developed as part of the Procedures, Patient Safety, and Point-of-Care Ultrasound (POCUS) rotation for our IM residency program — a university-based program with 95 categorical residents and four or five 4^th^-year chief residents annually. The MPS operates at a 462-bed Veterans Affairs (VA) hospital and 716-bed university-affiliated county teaching hospital.

## PROGRAM DESCRIPTION

All categorical medicine interns were required to complete the 4-week Procedures, Patient Safety, and POCUS rotation. Each 4-week block had 2–3 participating interns and IM chief residents supervising the MPS on a rotating weekly basis. The MPS was available Monday through Friday from 8 am to 5 pm, excluding holidays, and performed core ultrasound-guided procedures including paracentesis, thoracentesis, lumbar puncture, knee arthrocentesis, and internal jugular vein catheterization (non-tunneled, non-hemodialysis catheters). At the beginning of each rotation, chief residents oriented the incoming interns to the MPS and the process for logging procedures. The chief residents trained the interns in ultrasound-guided procedures through a combination of didactics and simulation-based procedure practice on task trainers. Didactics reviewed details of the procedures performed by the MPS, including the indications, contraindications, benefits, risks, and procedure steps with ultrasound guidance.

All procedures were performed by interns and directly supervised by a chief resident. The performing intern or supervising chief resident was responsible for logging all consults (performed and deferred) into the procedure log. The procedure log was a shared spreadsheet (Microsoft^®^ Excel^®^ 2011) stored on the local hospital network from 2011 to 2019, followed by REDCap™ (Vanderbilt University, Nashville, TN, USA) from 2019 to 2022. Every week, the MPS team had dedicated time to log procedure data and track delayed complications (24 h post-procedure). During this weekly meeting, the entire team gathered around computers and reviewed the past week’s procedures. Additionally, the MPS director reviewed the procedure logs up to twice per month to reconcile data and review complications.

From May to June of each academic year, the director of the MPS trained and signed off the incoming chief residents to lead the service independently. Training received by the chief residents included a combination of lectures, simulation-based procedure practice, and supervised procedure performance on patients with the MPS director at the bedside. For the remainder of the academic year (July to mid-May), the chief residents staffed the MPS independently and the director served as backup to address any questions about procedure consults.

Other components of the rotation included education on quality improvement methodology and attending root cause analyses, mock codes, and diagnostic cardiac and pulmonary POCUS workshops. Up to one half-day per week was dedicated to these other components.

### Resources Required

Resources required for the chief resident MPS are summarized in ESM Appendix 1. The faculty director was a hospitalist with expertise in diagnostic and procedural POCUS. Responsibilities of the director included chief resident training and guidance; coordinating the procedure simulation sessions and diagnostic POCUS workshops; reviewing the procedure log and complications associated with the MPS; and serving as a liaison to the hospitals for issues regarding ultrasound and procedural equipment, documentation, and policies.

The IM residency program had four or five 4^th^-year chief residents each year who received junior faculty appointments. Responsibilities of the chief residents specific to the procedure rotation included staffing the MPS, training the categorical medicine interns, maintaining the procedure log, and tracking complications. The Chief Resident in Quality and Patient Safety provided separate instruction on quality improvement methodology and coordinated the root cause analyses and mock codes.

## PROGRAM EVALUATION

We performed a retrospective observational study from 2011 to 2022 of all procedures (paracentesis, thoracentesis, lumbar puncture, knee arthrocentesis, and internal jugular vein catheterization) performed by our MPS at a VA hospital and university-affiliated county teaching hospital. Data were collected from the procedure logs including number of procedure consults, procedure attempts, success rates, and minor and major complications (immediate and within 24 h). Major complications were defined based on prior literature.^5–9^ All interns received a post-rotation survey to obtain both quantitative and qualitative feedback. This study was reviewed by the Investigational Review Board of the University of Texas Health San Antonio and was deemed to be non-research (Protocol Number: 20220913NRR).

**Figure 1:**
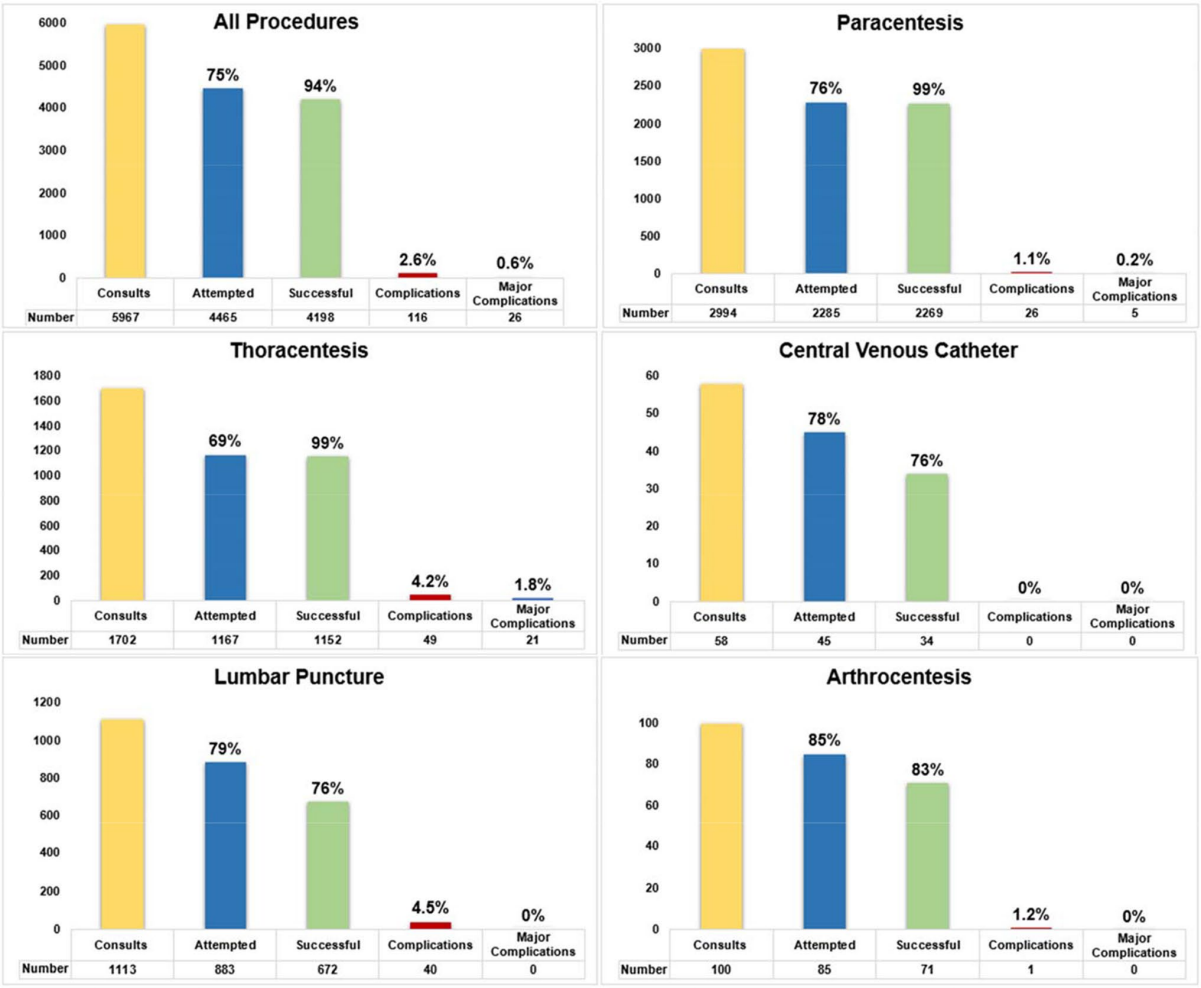
Bedside procedure consults and outcomes of a 4^th^-year internal medicine chief resident medical procedure service from 2011 to 2022. Note: Central venous catheter procedures were insertion of non-tunneled, non-hemodialysis catheters in the internal jugular vein and arthrocentesis was performed on knees only.

Between 2011 and 2022, 320 categorical IM interns and 48 chief residents participated in the MPS. A total of 5967 procedure consults were received and 4465 (75%) consults resulted in a procedure attempt. The remaining 1502 (25%) consults were deferred due to insufficient fluid volume to drain, procedure contraindication, patient declining the procedure, change in management plan by primary team, or other reason. The overall procedure success rate was 94% and overall complication rate was 2.6%. The major complication rate was 0.6% (Fig. 1 and Table 1). Success, complication, and major complication rates for paracentesis (*n*=2285) were 99%, 1.1%, and 0.2%, respectively; 99%, 4.2%, and 1.8% for thoracentesis (*n*=1167); 76%, 4.5%, and 0% for lumbar puncture (*n*=883); 83%, 1.2%, and 0% for knee arthrocentesis (*n*=85); and 76%, 0%, and 0% for internal jugular vein catheterization for non-tunneled central line placement (*n*=45).

**Table 1:** Procedural Complications of a Medical Procedure Service led by 4^th^-Year Internal Medicine Chief Residents from 2011 to 2022

Complications		
**Paracentesis**	***N***	**%**
**Overall**	**26**	**1.1**
Bleeding	12	0.53
Minor*	10	0.44
* Hemoperitoneum†*	2	0.09
Ascites leak	10	0.44
Hypotension†	3	0.13
Pain	1	0.04
**Thoracentesis**	***N***	**%**
**Overall**	**49**	**4.2**
Pneumothorax†	16	1.37
* Chest tube required*	2	0.17
Pain	16	1.37
Bleeding	7	0.60
Minor*	4	0.34
* Hemothorax†*	2	0.17
* Other major†*	1	0.09
Cough or dyspnea	3	0.26
Re-expansion pulmonary edema†	2	0.17
Other	5	0.43
**Lumbar puncture**	***N***	**%**
**Overall**	**40**	**4.5**
Post-dural puncture headache	28	3.17
Bleeding	5	0.57
Minor*	5	0.57
Pain	5	0.57
Other	2	0.23
**Knee arthrocentesis**	***N***	**%**
**Overall**	**1**	**1.2**
Bleeding	0	0
Pain	1	1.2
**Central venous catheter‡**	***N***	**%**
**Overall**	**0**	**0**
Bleeding	0	0
Pneumothorax†	0	0

*Minor bleeding: no transfusion or intervention required

†Major complication

‡Non-tunneled, non-hemodialysis catheters in internal jugular vein

On a 5-point Likert scale, the rotation received mean ratings of 4.6 and 4.3 representing good to excellent for overall learning quality and opportunity to perform required procedures, respectively. In qualitative feedback, interns reported that they received quality teaching and supervision by the chief residents, ample procedure opportunities with some variability in day-to-day procedure consults, and improvement in their comfort and proficiency in bedside procedures with ultrasound guidance.

## DISCUSSION

We have demonstrated that 4^th^-year IM chief residents trained in ultrasound-guided procedures can safely lead an MPS with high procedure success rates and low complication rates. The procedure rotation was highly rated by interns who valued the training and supervision received by the chief residents. Clinical outcomes of our chief resident–led MPS were comparable to those reported in a 2021 meta-analysis of 8 predominantly attending physician–led procedure services that reported pooled success and complication rates of 94.7% and 2.1%, respectively.^2^ Similarly, our major complication rate of 0.6% was comparable to a recent MPS study reporting 0.8%.^5^

Our success rate of 76% for lumbar puncture was lower than that of paracentesis and thoracentesis, but reassuringly this outcome is comparable to two recent studies of MPS’s supervised by attending physicians which reported success rates ranging from 73 to 79.4%.^5,10^ Factors associated with failure to obtain cerebrospinal fluid, including obesity and prior spinal surgery, were frequently encountered in our patient population.^10,11^ Also, in our local hospital practices, it is typically recommended that the primary teams or the MPS evaluate and attempt to perform a lumbar puncture at the bedside prior to referring the patient to interventional radiology. Therefore, attempting lumbar punctures on patients with known characteristics lowering the probability of a successful procedure, such as morbid obesity or prior spinal surgery, likely contributed to the MPS’s lower success rate. For central venous catheterization and arthrocentesis, the lower relative success rates may have been driven by the low volume of these procedures. A sufficient volume of procedures is essential to enhance procedural skills and achieve favorable success and complication rates.^4,12–14^

The structure and curricular components of our MPS are similar to other attending physician–led MPS’s with respect to availability during weekdays and business hours only, types of procedures performed, and inclusion of both didactic and simulation-based training methods.^1,3,15–18^ However, a chief resident–led MPS is a novel approach to overcome the limited availability of experienced attending physicians who can serve as proceduralists to establish an MPS. Our unique staffing model may allow other IM residency programs to gain the potential benefits of creating an MPS which include improvements in procedural volume, patient throughput, procedural education of trainees, and healthcare costs.^3^ Most MPS’s described in the literature include hospitalists, pulmonologists, or critical care attendings as the supervising proceduralists.^3^ One notable exception is the study by Gorgone et al. which described an MPS independently run by residents who perform most procedures unsupervised, but could seek faculty supervision when needed.^4^ Our MPS differed in that our 4^th^-year chief residents received junior faculty appointments and supervised nearly all intern-performed procedures without additional faculty support.

Strengths of our study include a high number of procedures performed (*n*=4465) and long study duration (>10 years) compared to prior studies ranging from 2 to 60 months.^3^ Our study also provides quantitative and qualitative data on MPS complications by procedure type, as few MPS studies have previously provided this data.^1,2,18–20^

Our study has several limitations. First, as a single-center study our results may have limited external validity to other healthcare systems. Second, though the complication rates for arthrocentesis and central venous catheterization were low, these results may not be reproducible given the low number of these procedures performed. However, we chose to include these data as they may guide resource allocation for training and equipment for institutions seeking to establish an MPS. Given the relatively infrequent consults for arthrocentesis and central line placement, some procedure services may choose to prioritize the more common procedures, namely paracentesis, thoracentesis, and lumbar puncture, especially when starting a new service. Third, lack of standardized definitions for certain procedural complications, such as pain, may have led to variability in reporting by the MPS team. Further, though scheduled time was provided for procedure log data entry and the MPS director reviewed the procedure log up to twice per month to reconcile data, we cannot rule out the possibility of incomplete or missing data which may affect the results. We also cannot rule out the possibility of bias by the participants to underreport complications, but this is less likely because all data were originally collected for quality assurance and performance improvement.^21^ Finally, we followed patients for 24 h for delayed complications, whereas some studies monitored patients for more than 24 h.^6,18,20^ While 24 h is likely sufficient to detect most delayed complications, this was likely insufficient time to detect central line–associated blood stream infections, and this complication was not explicitly tracked.

In conclusion, an MPS led by 4^th^-year IM chief residents is a practical yet safe approach for IM residency programs to establish an MPS when attending physicians with expertise in ultrasound-guided procedures are not available. Our chief resident–led MPS had procedure success and complication rates similar to attending physician–led procedure services reported in the literature. Future studies shall investigate health services outcomes, such as impact on length of stay, costs, and patient experience, when procedures are performed by a chief resident–led MPS versus traditional approaches.

### Supplementary Information

ESM 1 (DOCX 15 kb)
