# Supplementary material for: Development of a Chief Resident Medical Procedure Service: a 10-Year Experience
Source: J Gen Intern Med. 2023 May 26;38(13):3077–81. doi: 10.1007/s11606-023-08234-z (PMC10593632; doi:10.1007/s11606-023-08234-z)
Supplement: Supplementary file 1 — (DOCX 15 kb) [file 11606_2023_8234_MOESM1_ESM.docx]

**Appendix 1. Resources Required for a Chief-Resident Medical Procedure Service**

| **Resource** | **Details** |
| --- | --- |
| Faculty Director | - Faculty with expertise in ultrasound-guided procedures (~25% FTE) - Responsibilities:   - Train and sign-off the new chief residents (6-7 weeks/year)   - Coordinate intern procedure simulation and POCUS teaching activities   - Serve as backup to the chief residents for consult questions   - Review and reconcile procedure log data including review of complications associated with the MPS   - Serve as a liaison to the hospitals for issues pertaining to ultrasound equipment, documentation, and policies |
| Chief Residents | - 4-5 IM chief residents per year with junior-faculty appointments - Responsibilities:   - Staff the MPS and supervise interns in ultrasound-guided procedures, rotating on a weekly basis   - Lead procedure simulation training   - Maintain the procedure log and complication tracking   - Provide instruction on quality improvement methodology and coordinate root cause analyses and mock codes* |
| Ultrasound Equipment | - Ultrasound machine(s)   - Cart-based system ($20-50K)   - Handheld device ($4-10K) - Transducers†   - Linear-array   - Phased-array   - Image transfer capabilities |
| Procedure Simulation Equipment | - Procedure task trainers (~$15K for 1 set of paracentesis, thoracentesis, IJV central line, lumbar puncture, knee arthrocentesis) - Ultrasound machines (either dedicated or shared) - Procedure kits and needles/syringes for paracentesis/thoracentesis, central line, lumbar puncture, knee arthrocentesis |

*The designated Chief Resident in Quality and Patient Safety is responsible for these tasks †Need linear and phased-array transducers at minimum but having a curvilinear transducer can be advantageous for some diagnostic and procedural applications
FTE, full-time equivalent; POCUS, point-of-care ultrasound; MPS, medical procedure service; IM, internal medicine; IJV, internal jugular vein
